# Reliability of ankle dorsiflexor muscle strength, rate of force development, and tibialis anterior electromyography after stroke

**DOI:** 10.12688/f1000research.132415.1

**Published:** 2023-04-20

**Authors:** Sharon Olsen, Denise Taylor, Imran Khan Niazi, Grant Mawston, Usman Rashid, Gemma Alder, Verna Stavric, Rasmus Bach Nedergaard, Nada Signal

**Affiliations:** 1 Health and Rehabilitation Research Institute, Auckland University of Technology, Auckland, New Zealand; 2 Centre for Chiropractic Research, New Zealand College of Chiropractic, Auckland, New Zealand; 3 Department of Health Science and Technology, Aalborg University, Aalborg, Denmark; 4 Mech-Sense, Aalborg University Hospital, Aalborg, Denmark; 5 Department of Clinical Medicine, Aalborg University, Aalborg, Denmark

**Keywords:** muscle strength, maximal voluntary contraction, muscle power, rate of force development, electromyogram, outcome measure, reliability, stroke

## Abstract

Background: Measures of hemiparetic ankle dorsiflexor muscle strength and rate of force development (RFD) are often used to determine the efficacy of rehabilitation interventions after stroke. However, evidence supporting the reliability of these measures is limited. This brief report provides a secondary analysis investigating the between-session reliability of isometric ankle dorsiflexor muscle strength, rate of force development (RFD), and tibialis anterior electromyography (TA EMG), in people with chronic stroke.

Method: Participants (n=15) completed three maximal isometric contractions of the ankle dorsiflexor muscles as fast as possible using a rigid dynamometer. Tests were repeated seven days later. Outcomes included ankle dorsiflexor isometric maximal voluntary contraction (MVC), RFD in the first 200ms (RFD200ms), time to reach 90% MVC, and peak TA EMG. Data were analysed for 13 participants using intra-class correlation coefficients (ICC) and standard error of the measure (SEM).

Results: When the mean of three trials was analysed, there was excellent reliability for isometric dorsiflexor MVC (ICC 0.97,95% CI 0.92 to 0.99), moderate reliability for TA EMG (ICC 0.86, 95% CI 0.60 to 0.96) and time to reach 90% MVC (ICC 0.8, 95% CI 0.53 to 0.93]) and poor reliability for dorsiflexor RFD200ms (ICC 0.79, 95% CI 0.48 to 0.92]).

Conclusion: Given the functional significance of the ankle dorsiflexors, future research should investigate more reliable methods for measuring rapid force production in the dorsiflexor muscles after stroke.

## Introduction

Ankle dorsiflexor impairments are common after stroke ^1^ affecting both muscle strength, the force exerted during a single maximal effort, and muscle power, the ability to exert force over a short time. ^2^ Impaired strength of the hemiparetic dorsiflexor muscles is associated with reduced walking endurance, ^3^ walking speed, ^4^ and functional mobility. ^1^ Impaired dorsiflexor muscle power or rapid force production may limit the ability to react quickly during perturbations ^5^^,^^6^ and contribute to falling. ^7^ Measures of dorsiflexor strength, power, and rate of force development (RFD), are commonly used to determine the efficacy of rehabilitation interventions ^8^^,^^9^ and thus, their reliability should be considered.

Muscle strength can be measured isokinetically or isometrically through a maximal voluntary contraction (MVC) using rigid gold-standard dynamometry. ^10^^,^^11^ Between-session reliability of isokinetic dorsiflexor MVCs in the hemiparetic limb ranges from moderate to excellent with ICCs ranging from 0.84 [95% CI (confidence interval) 0.52 to 0.96] ^12^ to 0.98. ^13^ However, isokinetic testing requires the ability to dorsiflex through full range at a given speed, thus excluding those with more severe stroke who are unable to do so. ^14^ Isometric dorsiflexor MVCs, tested with rigid dynamometry, can be recorded in people with more severe hemiparesis, but have demonstrated only moderate between-session reliability (ICC 0.71). ^15^ Alongside dorsiflexor MVC measures, it is common to concurrently record surface electromyography (EMG) of the tibialis anterior (TA) as a measure of motor unit activity. While TA EMG peak amplitude has been shown to be highly reliable in healthy adults within a session, ^16^ it’s between-session reliability after stroke is only moderate (ICC 0.67). ^15^

Rapid force production and muscle power in the hemiparetic limb has been measured using several outcomes ^6^^,^^17^^–^^19^ including the RFD. The between-session reliability of dorsiflexor peak RFD measured with hand-held dynamometry (HHD) was good (ICC 0.92, 95% CI 0.83 to 0.96) ^20^ in people with stroke who could walk unaided. However, this HHD method had poor concurrent validity against gold-standard dynamometry, ^21^ suggesting the reliability of RFD should be assessed using a rigid dynamometry system.

To address these limitations, this brief report will provide a reliability analysis that was performed on a dataset from an experimental study, where baseline measures of ankle dorsiflexor strength and RFD were collected twice, seven days apart. ^9^ This analysis aimed to determine the between-session reliability of isometric ankle dorsiflexor MVC, ankle dorsiflexor muscle RFD in the first 200ms (RFD200ms), time to reach 90% peak force, and TA EMG, in people with chronic stroke.

## Methods

### Study design

This observational study utilised baseline measurement data that had been collected in an experimental study. ^9^ Baseline measures were collected on two occasions, seven days apart.

### Setting

The study was conducted in a research laboratory at the Auckland University of Technology, Auckland, New Zealand.

### Participants

The 15 participants were adults, more than 6 months post stroke, with hemiparesis affecting ankle dorsiflexion. The sample size was based on that required for the broader experimental study. ^9^ Exclusion criteria were significant cognitive/perceptual/communication deficits, cerebellar stroke, absent ankle dorsiflexor force, or medical conditions that would impact safety or protocol completion. ^9^ Written informed consent, ethical approval (Health and Disability Ethics Committees 17/NTB/80), and trial registration were completed (ACTRN12617000838314).

### Measurement outcomes

The measurement outcomes were: isometric ankle dorsiflexor peak MVC, ankle dorsiflexor muscle RFD in the first 200ms (RFD200ms), time to reach 90% MVC (Time to 90% MVC), and peak TA EMG.

### Measurement procedures

Detailed procedures have been published elsewhere. ^9^^,^^22^ Participants sat with their hemiparetic leg in a rigid purpose-built ankle dorsiflexion/plantarflexion dynamometer with the foot plate angled 25° into plantarflexion, knee flexion ≈50°, straps/guards at the hips, knee, ankle, metatarsals and toes, ^23^ and EMG electrodes over the TA muscle in accordance with SENIAM guidelines ( seniam.org). Following two submaximal practices, participants performed three 4-5 second isometric dorsiflexor MVCs (2 minutes between each). Participants were instructed to “pull as fast and hard as possible” and received loud verbal encouragement and real-time visual feedback. Force signals were amplified (×200, 500, or 1000 depending on amplitude) (Forza, OT Bioelettronica, Italy). EMG data was amplified (×500) (AMT-8, Bortec Biomedical, Canada). Force and EMG data were sampled at 1961Hz using a data acquisition board (Micro 1401, CED, UK) and Spike2 software (CED, UK). Procedures were replicated for the second session.

### Data processing

MVC amplitudes ^15^ were calculated as the difference between the mean baseline signal (500ms window) and the peak amplitude, in Spike2 software (CED, UK). For other measures, data was exported into LabVIEW 2017 software (National Instruments, United States) and the force data was filtered using a zero-phase shift 15 Hz low-pass 4 ^th^ order filter. ^21^^,^^24^ Movement onset was automatically identified where the signal exceeded the mean baseline signal by 3 SDs, and then confirmed visually. The baseline window and the onset threshold could be individualised to ensure the onset was identified correctly for each contraction. RFD200ms ^24^^,^^25^ was determined by dividing force at 200ms by time. Time taken to reach 90% of peak force was also determined. ^18^^,^^26^ TA EMG data was band-pass filtered (10–500 Hz). The root mean square (RMS) of the EMG signal was calculated 1-s either side of the peak force, and peak amplitude ^16^ of the RMS signal was determined. All measures were calculated for each of the three contractions, then exported into Microsoft Excel (version 16.35, Microsoft Corporation, US) where the mean of three trials and the best of three trials were calculated.

### Statistical analysis

Data were imported into R for reliability analysis (R version 4.1.1 ^27^). Data normality was evaluated with the Shapiro-Wilk test. The intra-class correlation coefficient (ICC (2, 1), absolute agreement) from a 2-way random effects model was calculated, as were the standard error of measurement (SEM) and the SEM%. Correlation coefficients were interpreted as excellent (≥ 0.90), good (0.75–0.89), moderate (0.50–0.74) and poor (<0.50) based on their lower bound 95% CIs. ^28^

## Results

### Participants

Data for two participants were excluded due to failure to correctly complete the protocol. Therefore, the analysis included 13 participants (male n=6, mean age 68.5±10.6 years, mean 6.0±5.4 years post-stroke, left hemiparesis n=10). EMG data was missing for one further participant. Participants presented with a range of lower limb weakness.

### Reliability analysis

The reliability analysis is reported in Table 1. MVC measures were the most reliable, with the mean of three trials displaying higher reliability than the best of three trials. TA EMG data demonstrated moderate reliability. For measures of rapid force production, when using the mean of three trials, the Time to 90% MVC had moderate reliability and RFD200ms had poor reliability; both measures demonstrated very low lower-bound CIs when only the best trial was analysed ( Table 1).

**Table 1.** Reliability of all outcomes between test 1 and test 2.

	Test 1	Test 2	ICC (2,1) [95% CI]	SEM	SEM%	Absolute agreement
**MVC** ^**MEAN**^ **(N)**	139±65	145±66	0.97 [0.92, 0.99]	10	7	Excellent
**MVC** ^**BEST**^ **(N)**	146±67	151±66	0.97 [0.90, 0.99]	12	8	Excellent
**TA EMG** ^**MEAN**^ **(V)**	0.19±0.13	0.18±0.12	0.86 [0.60, 0.96]	0.05	25	Moderate
**TA EMG** ^**BEST**^ **(V)**	0.21±0.14	0.19±0.12	0.86 [0.60, 0.96]	0.05	23	Moderate
**RFD200ms** ^**MEAN**^ **(N/s)**	267±160	246±123	0.79 [0.48, 0.92]	65	24	Poor
**RFD200ms** ^**BEST**^ **(N/s)**	313±198	297±137	0.61 [0.17, 0.86]	106	34	Poor
**Time to 90% MVC** ^**MEAN**^ **(s)**	1.46±0.76	1.64±0.82	0.80 [0.53, 0.93]	0.3	23	Moderate
**Time to 90% MVC** ^**BEST**^ **(s)**	2.04±1.14	2.33±1.45	0.52 [0.00, 0.82]	0.9	44	Poor

All outcomes displayed a normal distribution. Descriptive statistics are presented as mean±SD.

Abbreviations: MVC, maximal voluntary contraction; MEAN, mean of 3 trials; BEST, best of 3 trials; TA EMG, tibialis anterior electromyography; RFD200ms, rate of force development in the first 200ms; Time to 90% MVC, time to reach 90% of peak maximal voluntary contraction.

## Discussion

This is the first study to show excellent between-session reliability of isometric (rather than isokinetic) dorsiflexor MVCs in people with stroke (MVC ^MEAN^ ICC 0.97, 95% CI 0.92 to 0.99). Our results were comparable with those of Eng *et al.* ^13^ using an isokinetic MVC. Importantly, the isometric method proposed here can be applied to people with more severe lower limb weakness. Our MVC reliability results were superior to the isometric MVC results of Klarner and colleagues who found moderate between-session reliability for hemiparetic dorsiflexor MVCs over three sessions (ICC 0.71). ^15^ They analysed the best of only two trials, rather than the three trials used in this study, and did not describe any system to strap the toes as recommended to reduce measurement variability ^23^; this may have lowered their ICC. Our reliability findings for TA EMG, which represent motor unit recruitment at the peak of the MVC, demonstrated only moderate reliability (TA EMG ^MEAN^ ICC 0.86, 95% CI 0.60 to 0.96), suggesting that EMG is prone to greater biological and/or measurement variability than force measures. As with the MVC data, our TA EMG data appeared more reliable than that previously reported (ICC 0.67). ^15^

This study is also the first to report on the reliability of RFD or rapid force production of the hemiparetic dorsiflexor muscles using a rigid dynamometer. The ICCs were deemed moderate for Time to 90% MVC ^MEAN^ (ICC 0.80, 95% CI 0.53 to 0.93) and poor for RFD200ms ^MEAN^ (ICC 0.79, 95% CI 0.48 to 0.92). These findings were inferior to those of Mentiplay and colleagues who used HHD to measure hemiparetic dorsiflexor RFD (ICC 0.92, 95% CI 0.83 to 0.96, n=28). ^20^ Several factors may have contributed to these contrasting findings. Mentiplay *et al’s* participants could walk unaided, whereas our sample had variable lower limb impairment; they also measured the ankle in neutral, ^20^ whereas we positioned the ankle in ≈25° plantarflexion based on the optimum position for producing dorsiflexion force ^29^ and reducing the impact of antagonist muscle tone. ^24^ Data processing methods also differed between the studies. Mentiplay and colleagues HHD method sampled force data at only 40 Hz, ^20^^,^^21^ much lower than recommended, ^24^^,^^30^ and then interpolated this to equate 1000 Hz, which may have increased reliability. Our study analysed RFD in the first 200ms, whereas Mentiplay *et al.* ^20^ scanned successive 200ms windows to find the peak RFD, a method that excludes movement onset and any associated artefacts or issues with identifying onset. ^21^ This very early force generation is particularly relevant for people with stroke who have lower motor unit discharge rates ^6^^,^^31^ and may be more functionally important than maximal muscle strength or power, especially under circumstances where a rapid response is required (e.g., to prevent falling). ^32^ Thus, while measuring RFD later in the movement may be more reliable, ^20^^,^^21^ this measure lacks ecological validity. This concern is supported by the poor concurrent validity of the HHD RFD method against gold-standard dynamometry. ^21^ Given our findings, further research is needed to explore more reliable methods for measuring hemiparetic RFD and muscle power that better account for sources of biological and measurement tool variability.

## Conclusions

This analysis demonstrated excellent between-session reliability for hemiparetic dorsiflexor isometric MVC and moderate reliability for hemiparetic TA EMG. Dorsiflexor muscle RFD measures had poor to moderate reliability. Future research should investigate more reliable tools for measuring hemiparetic dorsiflexor muscle RFD and muscle power. Given the significance of dorsiflexor muscle function to lower limb recovery after stroke, it is vital this aspect of muscle function is better understood to enable more targeted rehabilitation.

### Ethical considerations

The study was conducted in accordance with the Declaration of Helsinki, and approved by the Health and Disability Ethics Committees (17/NTB/80).

## Data Availability

Ethical approval for data sharing has not been obtained. Requests for access to the data can be made to the corresponding author by providing the reason for the request and the benefits of data sharing, so that ethical approval can be sought.
