# Reliability of ankle dorsiflexor muscle strength, rate of force development, and tibialis anterior electromyography after stroke

**DOI:** 10.12688/f1000research.132415.2

**Published:** 2024-06-04

**Authors:** Sharon Olsen, Denise Taylor, Imran Khan Niazi, Grant Mawston, Usman Rashid, Gemma Alder, Verna Stavric, Rasmus Bach Nedergaard, Nada Signal

**Affiliations:** 1 Health and Rehabilitation Research Institute, Auckland University of Technology, Auckland, New Zealand; 2 Department of Health Science and Technology, Aalborg University, Aalborg, Denmark; 3 Centre for Chiropractic Research, New Zealand College of Chiropractic, Auckland, New Zealand; 4 Department of Clinical Medicine, Aalborg University, Aalborg, Denmark; 5 Mech-Sense, Aalborg University Hospital, Aalborg, Denmark

**Keywords:** muscle strength, maximal voluntary contraction, muscle power, rate of force development, electromyogram, outcome measure, reliability, stroke

## Abstract

**Background:**

Measures of hemiparetic ankle dorsiflexor muscle strength and rate of force development (RFD) are often used to determine the efficacy of rehabilitation interventions after stroke. However, evidence supporting the reliability of these measures is limited. This brief report provides a secondary analysis investigating the between-session reliability of isometric ankle dorsiflexor muscle strength, rate of force development (RFD), and tibialis anterior electromyography (TA EMG), in people with chronic stroke.

**Method:**

Participants (n=15) completed three maximal isometric contractions of the ankle dorsiflexor muscles as fast as possible using a rigid dynamometer. Tests were repeated seven days later. Outcomes included ankle dorsiflexor isometric maximal voluntary contraction (MVC), RFD in the first 200ms (RFD200ms), time to reach 90% MVC, and peak TA EMG. Data were analysed for 13 participants using intra-class correlation coefficients (ICC) and standard error of the measure percentage (SEM%).

**Results:**

Reliability was higher when analysing the mean of three trials rather than the best of three trials. There was excellent reliability for isometric dorsiflexor MVC (ICC 0.97 [95% CI 0.92, 0.99], SEM% 7%). However, for other outcomes, while the ICC indicated good reliability, the lower bound of the 95% confidence interval of the ICC fell in the moderate range for TA EMG (ICC 0.86 [95% CI 0.60, 0.96], SEM% 25%) and time to reach 90% MVC (ICC 0.8 [95% CI 0.53, 0.93], SEM% 23%) and in the poor range for dorsiflexor RFD200ms (ICC 0.79 [95% CI 0.48, 0.92], SEM% 24%).

**Conclusion:**

The findings raise concerns about the reliability of measures of rapid force production in the dorsiflexor muscles after stroke. Given the functional significance of the ankle dorsiflexors, larger studies should be conducted to further investigate these concerns and explore reliable methods for measuring rapid force production in the hemiparetic dorsiflexor muscles.

## Introduction

Ankle dorsiflexor impairments are common after stroke ^1^ affecting both muscle strength (the force exerted during a single maximal effort) and muscle power (the ability to exert force over a short time). ^2^ Impaired strength of the hemiparetic dorsiflexor muscles is associated with reduced walking endurance, ^3^^,^^4^ walking speed, ^1^^,^^5^^,^^6^ functional mobility, ^7^ and community integration. ^4^ Impaired dorsiflexor muscle power or rapid force production may limit the ability to react quickly during perturbations ^8^^,^^9^ and contribute to falling. ^10^^,^^11^ Rapid force production requires recruitment of a large number of motor units as well as a high motor unit firing frequency, both of which are impaired in the hemiparetic dorsiflexor muscles after stroke. ^9^ This results from central deficits, which reduce neural input to the motor neuron pool, but is also limited by peripheral changes, such as the reduction in the size of type 2a muscle fibres in the hemiparetic tibialis anterior ^12^ and soft tissue stiffness and spasticity in the antagonist plantarflexor muscles. ^13^ Due to their key role in lower limb function, measures of dorsiflexor muscle strength, power, and rate of force development (RFD), are commonly used to determine the efficacy of rehabilitation interventions ^14^^,^^15^ and thus, their reliability should be considered.

Muscle strength can be measured isokinetically or isometrically through a maximal voluntary contraction (MVC) using rigid gold-standard dynamometry. ^16^^,^^17^^,^^18^ Isokinetic MVCs involve muscle contraction against accommodating resistance through the joints range of movement at a constant velocity, whereas isometric MVCs involve muscle contraction against stationary resistance at a set joint angle. ^19^ Between-session reliability of isokinetic dorsiflexor MVCs in the hemiparetic limb ranges from moderate to excellent with ICCs ranging from 0.84 [95% CI (confidence interval) 0.52 to 0.96] ^20^ to 0.98. ^21^ However, isokinetic testing requires the ability to dorsiflex through full range at a given speed, thus excluding those with more severe stroke who are unable to do so. ^22^ Isometric dorsiflexor MVCs, tested with rigid dynamometry, can be recorded in people with more severe hemiparesis, but have demonstrated only moderate between-session reliability (ICC 0.71). ^23^ Alongside dorsiflexor MVC measures, it is common to concurrently record surface electromyography (EMG) of the tibialis anterior (TA) as a measure of motor unit activity. While TA EMG peak amplitude has been shown to be highly reliable in healthy adults within a session, ^24^ it’s between-session reliability after stroke is only moderate (ICC 0.67). ^23^

Rapid force production and muscle power in the hemiparetic limb has been measured using several outcomes ^9^^,^^25^^–^^27^ including the RFD. The between-session reliability of dorsiflexor peak RFD measured with hand-held dynamometry (HHD) was good to excellent (ICC 0.92, 95% CI 0.83 to 0.96) ^28^ in people with stroke who could walk unaided. However, this HHD method had poor concurrent validity against gold-standard dynamometry, ^29^ suggesting the reliability of RFD should be assessed using a rigid dynamometry system.

To address these limitations, this brief report will provide a reliability analysis that was performed on a dataset from an experimental study, where baseline measures of ankle dorsiflexor strength and RFD were collected twice, seven days apart. ^15^ This analysis aimed to determine the between-session reliability of isometric ankle dorsiflexor MVC, ankle dorsiflexor muscle RFD in the first 200ms (RFD200ms), time to reach 90% peak force, and TA EMG, in people with chronic stroke.

## Methods

### Study design

This observational study utilised baseline measurement data that had been collected in an experimental study. ^15^ Baseline measures were collected on two occasions, seven days apart. The null hypothesis was that the outcome measures are not reliable (intra-class correlation coefficient (ICC) < 0.5).

### Setting

The study was conducted in a research laboratory at the Auckland University of Technology, Auckland, New Zealand.

### Participants

The 15 participants were adults, more than 6 months post stroke, with hemiparesis affecting ankle dorsiflexion movement. The sample size was based on that required for the broader experimental study. ^15^ Exclusion criteria were significant cognitive/perceptual/communication deficits, cerebellar stroke, inability to produce ankle dorsiflexor force against the dynamometer, or medical conditions that would impact safety or protocol completion. ^15^ Written informed consent, ethical approval (Health and Disability Ethics Committees 17/NTB/80), and trial registration were completed (ACTRN12617000838314).

### Measurement outcomes

The measurement outcomes were: isometric ankle dorsiflexor peak MVC, ankle dorsiflexor muscle RFD in the first 200ms (RFD200ms), time to reach 90% MVC (Time to 90% MVC), and peak TA EMG.

### Measurement procedures

Detailed procedures have been published elsewhere. ^15^^,^^30^ Participants sat with their hemiparetic leg in a rigid purpose-built ankle dorsiflexion/plantarflexion dynamometer with the foot plate angled 25° into plantarflexion, knee flexion ≈50°, straps/guards at the hips, knee, ankle, metatarsals and toes, ^31^ and EMG electrodes over the TA muscle in accordance with SENIAM guidelines ( seniam.org). Following two submaximal practices, participants performed three isometric dorsiflexor MVCs; each lasted a duration of 4 to 5 seconds and a 2-minute rest was given between each MVC. Participants were instructed to “pull as fast and hard as possible” and received loud verbal encouragement and real-time visual feedback. Instructions were provided by the same researcher at both sessions. Force signals were amplified (×200, 500, or 1000 depending on amplitude) (Forza, OT Bioelettronica, Italy). EMG data was amplified (×500) (AMT-8, Bortec Biomedical, Canada). Force and EMG data were sampled at 1961Hz using a data acquisition board (Micro 1401, CED, UK) and Spike2 software (CED, UK). Procedures were replicated for the second session.

### Data processing

MVC amplitudes ^23^ were calculated as the difference between the mean baseline signal (500ms window) and the peak amplitude, in Spike2 software (CED, UK). For other measures, data was exported into LabVIEW 2017 software (National Instruments, United States) and the force data was filtered using a zero-phase shift 15 Hz low-pass 4 ^th^ order filter. ^29^^,^^32^ Movement onset was automatically identified where the signal exceeded the mean baseline signal by 3 SDs, and then confirmed visually by a single researcher. The baseline window and the onset threshold could be individualised by the researcher to ensure the onset was identified correctly for each contraction. RFD200ms ^32^^,^^33^ was determined by dividing force at 200ms by time. Time taken to reach 90% of peak force was also determined. ^26^^,^^34^ TA EMG data was band-pass filtered (10–500 Hz). The root mean square (RMS) of the EMG signal was calculated 1-s either side of the peak force, and peak amplitude ^24^ of the RMS signal was determined. All measures were calculated for each of the three contractions, then exported into Microsoft Excel (version 16.35, Microsoft Corporation, US) where the mean of three trials and the best of three trials were calculated.

### Statistical analysis

Data were imported into R for reliability analysis (R version 4.1.1 ^35^). Data normality was evaluated with the Shapiro-Wilk test. The intra-class correlation coefficient (ICC (2, 1), absolute agreement) from a 2-way random effects model was calculated, as were the standard error of measurement (SEM) and the SEM%. Correlation coefficients were interpreted as excellent (≥ 0.90), good (0.75–0.89), moderate (0.50–0.74) and poor (<0.50) based on their ICC and their lower bound 95% CI. ^36^^,^^37^

## Results

### Participants

Data for two participants were excluded due to failure to correctly complete the protocol; this was because one participant was not able to consistently follow the task instructions and another participant was observed falling asleep during the protocol. Therefore, the analysis included 13 participants (male n=6, mean age 68.5±10.6 years, mean 6.0±5.4 years post-stroke, left hemiparesis n=10). EMG data was missing for one further participant. Participants presented with a range of lower limb weakness, from mild to severe, and used a variety of outdoor mobility aids (unaided n = 4, quad or walking stick n = 5, walking frame n = 2, wheelchair n = 2) suggesting a range of walking abilities.

### Reliability analysis

The reliability analysis is reported in Table 1. MVC measures demonstrated excellent reliability, with the mean of three trials displaying slightly higher reliability (ICC 0.97 [95% CI 0.92, 0.99]) than the best of three trials (ICC 0.97 [95% CI 0.90, 0.99]). For TA EMG data, the ICCs were in the good range but the lower bound 95% CIs were in the moderate range (ICC 0.86 [95% CI 0.60, 0.06]). For measures of rapid force production, when using the mean of three trials, the Time to 90% MVC and RFD200ms had ICCs in the good range, but the lower bound 95% CIs were in the moderate range for Time to 90% MVC (ICC 0.80 [95% CI 0.53, 0.93]) and in the poor range for RFD200ms (ICC 0.0.79 [95% CI 0.48, 0.92]). Both measures of rapid force production demonstrated very low lower-bound CIs when only the best trial was analysed ( Table 1).

**Table 1.** Reliability of all outcomes between test 1 and test 2.

	Test 1	Test 2	ICC (2,1) [95% CI]	SEM	SEM%
**MVC**_**MEAN**_ **(N)**	139±65	145±66	0.97 [0.92, 0.99]	10	7
**MVC**_**BEST**_ **(N)**	146±67	151±66	0.97 [0.90, 0.99]	12	8
**TA EMG**_**MEAN**_ **(V)**	0.19±0.13	0.18±0.12	0.86 [0.60, 0.96]	0.05	25
**TA EMG**_**BEST**_ **(V)**	0.21±0.14	0.19±0.12	0.86 [0.60, 0.96]	0.05	23
**RFD200ms**_**MEAN**_ **(N/s)**	267±160	246±123	0.79 [0.48, 0.92]	65	24
**RFD200ms**_**BEST**_ **(N/s)**	313±198	297±137	0.61 [0.17, 0.86]	106	34
**Time to 90% MVC**_**MEAN**_ **(s)**	1.46±0.76	1.64±0.82	0.80 [0.53, 0.93]	0.3	23
**Time to 90% MVC**_**BEST**_ **(s)**	2.04±1.14	2.33±1.45	0.52 [0.00, 0.82]	0.9	44

All outcomes displayed a normal distribution. Descriptive statistics are presented as mean±SD.

Abbreviations: MVC, maximal voluntary contraction; MEAN, mean of 3 trials; BEST, best of 3 trials; TA EMG, tibialis anterior electromyography; RFD200ms, rate of force development in the first 200ms; Time to 90% MVC, time to reach 90% of peak maximal voluntary contraction.

## Discussion

This is the first study to show excellent between-session reliability of isometric (rather than isokinetic) dorsiflexor MVCs in people with stroke (MVC _MEAN_ ICC 0.97 [95% CI 0.92, 0.99]). Our results were comparable with those of Eng *et al.* ^21^ using an isokinetic MVC. Importantly, the isometric method proposed here can be applied to people with more severe lower limb weakness. Our MVC reliability results were superior to the isometric MVC results of Klarner and colleagues who found moderate between-session reliability (ICC 0.71) for hemiparetic dorsiflexor MVCs over three sessions, with a similar sample size (n=12). ^23^ They analysed the best of only two trials, rather than the three trials used in this study, and did not describe any system to strap the toes as recommended to reduce measurement variability ^31^; this may have lowered their ICC. Our reliability findings for TA EMG, which represent motor unit recruitment at the peak of the MVC, demonstrated lower reliability, with a good ICC and the lower bound 95% CI in the moderate range (TA EMG _MEAN_ ICC 0.86 [95% CI 0.60, 0.96). Alongside an SEM% of 23-25%, this suggests that TA EMG is prone to greater biological and/or measurement variability than peak force measures. Interestingly, as with the MVC data, our TA EMG data appeared more reliable than that previously reported (ICC 0.67) in sample of 12 people with chronic stroke. ^23^

This study is also the first to report on the reliability of RFD or rapid force production of the hemiparetic dorsiflexor muscles using a rigid dynamometer. While the ICCs were in the good range when three trials were analysed, the lower bound of the 95% CI of the ICCs indicated reliability could be only moderate for Time to 90% MVC _MEAN_ (ICC 0.80 [95% CI 0.53, 0.93]) and poor for RFD200ms _MEAN_ (ICC 0.79 [95% CI 0.48, 0.92]). These findings were inferior to those of Mentiplay and colleagues who used HHD to measure hemiparetic dorsiflexor RFD (ICC 0.92 [95% CI 0.83, 0.96], n=28). ^28^ Several factors may have contributed to these contrasting findings. Mentiplay *et al’s* participants could walk unaided, whereas our sample had variable lower limb impairment; they also measured the ankle in neutral, ^28^ whereas we positioned the ankle in ≈25° plantarflexion based on the optimum position for producing dorsiflexion force ^38^ and reducing the impact of antagonist muscle tone. ^32^ Data processing methods also differed between the studies. Mentiplay and colleagues HHD method sampled force data at only 40 Hz, ^28^^,^^29^ much lower than recommended, ^32^^,^^39^ and then interpolated this to equate 1000 Hz, which may have increased reliability. Our study analysed RFD in the first 200ms, whereas Mentiplay *et al.* ^28^ scanned successive 200ms windows to find the peak RFD, a method that excludes movement onset and any associated artefacts or issues with identifying onset. ^29^ This very early force generation is particularly relevant for people with stroke who have lower motor unit discharge rates ^9^^,^^40^ and may be more functionally important than maximal muscle strength or power, especially under circumstances where a rapid response is required (e.g., to prevent falling). ^41^ Thus, while measuring RFD later in the movement may be more reliable, ^28^^,^^29^ this measure may lack ecological validity. This concern is supported by the poor concurrent validity of the HHD RFD method against gold-standard dynamometry. ^29^ Given our findings, further research is needed to investigate these concerns about the reliability of rapid force production measures in the hemiparetic dorsiflexor muscles. This research should explore alternative methods for data collection and processing, ^32^ and seek to identify reliable methods for measuring hemiparetic RFD and muscle power that better account for sources of biological and measurement tool variability.

## Strengths and limitations

A key strength of this study was the application of an isometric MVC procedure that could be completed by people with more severe stroke. This enabled enrolment of a broad sample with a range of lower limb weakness and functional walking ability, increasing the generalisability of findings to a wider stroke population. Other methodological strengths included positioning of the ankle to optimise dorsiflexion force ^38^ and fixation of the toes to reduce measurement variability. ^31^ In addition, our approach to measuring RFD enabled evaluation of the very early force production (0-200ms) which has not been evaluated previously in the hemiparetic dorsiflexor muscles. Further research could explore the reliability of other time windows. The key limitation in this study was the sample size, which was below the n=30 recommended for reliability studies ^36^ but comparable with other studies in this field. ^20^^,^^21^^,^^23^ To address this limitation, we have been cautious with our interpretation of results, and considered both the ICC and its lower bound 95% CI ^36^^,^^37^ and have provided SEMs to enable comparisons with other literature. However, due to this limitation, it is recommended that the findings generated in this study are confirmed with further research in a larger sample.

## Conclusions

This analysis demonstrated excellent between-session reliability for hemiparetic dorsiflexor isometric MVCs. However, other measures of EMG and rapid force production were less reliable, with ICC 95% confidence intervals extending to the poor to moderate range, and SEM percentages between 23-25%. These findings, which utilises gold-standard dynamometry, raise concerns about the reliability of measures of rapid force production in the hemiparetic dorsiflexors muscles. Further research is required to examine reliability in a large sample of people stroke. In the meantime, researchers and clinicians should be cautious when interpreting rapid force production measures of the dorsiflexor muscles when determining the efficacy of stroke rehabilitation interventions. Given the significance of dorsiflexor muscle function to lower limb recovery after stroke, future research should investigate reliable tools for measuring hemiparetic dorsiflexor muscle RFD and muscle power. This will facilitate a greater understanding this aspect of muscle function and enable more targeted rehabilitation.

### Ethical considerations

The study was conducted in accordance with the Declaration of Helsinki, and approved by the Health and Disability Ethics Committees (17/NTB/80).

## Data Availability

Ethical approval for data sharing has not been obtained. Requests for access to the data can be made to the corresponding author by providing the reason for the request and the benefits of data sharing, so that ethical approval can be sought.
