# Supplementary material for: Reliability of ankle dorsiflexor muscle strength, rate of force development, and tibialis anterior electromyography after stroke
Source: F1000Res. 2026 Mar 4;12:423. Originally published 2023 Apr 20. [Version 3] doi: 10.12688/f1000research.132415.3 (PMC11359974; doi:10.12688/f1000research.132415.3)
Supplement: Peer review history

# Reviewer report: Reviewer response for version 2

**DOI:** 10.5256/f1000research.162800.r310093

**Published:** 2024-08-22

**Authors:** Ramachandran Sivakumar

**Affiliations:** 1 Sri Ramachandra Medical College and Research Institute, Chennai, Tamil Nadu, India

The study seems to be a offshoot of a larger study (?) based on the statement - "Detailed procedures have been published elsewhere".

 The MVC was produced using instruction to pull as fast and hard as possible, a loud verbal encouragement and real-time visual feedback. This procedure raises concern for generalizing the reproducibility. The encouragement and real time visual feedback are not common in clinical settings. 

 RFD is measured in first 200ms. Justification for duration window need to be added. This method has been mentioned as strength of the study.

 What is best value for 90% of peak force?, is the time taken in the trail with maximum peak force or trial with minimum time to reach 90% of peak force? In view of looking at ability to produce peak force to tackle real life scenarios requiring quick force production, the best timing must be the shortest time at which 90% force reached.  

 The reliability could also be influenced by difference in functional levels of the participants. 

 ICC used is appropriate, however, other statistical elements and presentations has to be commented by a statistician.

Is the work clearly and accurately presented and does it cite the current literature?

Yes

If applicable, is the statistical analysis and its interpretation appropriate?

Partly

Are all the source data underlying the results available to ensure full reproducibility?

Yes

Is the study design appropriate and is the work technically sound?

Partly

Are the conclusions drawn adequately supported by the results?

Yes

Are sufficient details of methods and analysis provided to allow replication by others?

Yes

Reviewer Expertise:

Stroke rehabilitation, Posture control assessment and training.

I confirm that I have read this submission and believe that I have an appropriate level of expertise to confirm that it is of an acceptable scientific standard, however I have significant reservations, as outlined above.

# Response

**Published:** 2026-02-26

**Authors:** Sharon Olsen

**Affiliations:** HRRI, Auckland University of Technology, Auckland, New Zealand

Apologies for the delay in providing an updated manuscript. We were waiting on feedback from another reviewer. 

**3.1 The MVC was produced using instruction to pull as fast and hard as possible, a loud verbal encouragement and real-time visual feedback. This procedure raises concern for generalizing the reproducibility. The encouragement and real time visual feedback are not common in clinical settings.**

 Thank you for this thoughtful comment. We agree that it is important to clarify the rationale for the motivational strategies used during MVC testing.

 Strong verbal encouragement during maximal strength testing is supported by evidence showing it increases peak isometric force output (McNair et al., 1996). Furthermore, instructions to “pull as hard and fast as you can” produce greater torque and power outputs than no instructions (Rendos et al., 2019). Thus, verbal encouragement is considered appropriate to maximise performance and can be reproduced in clinical settings.

 Visual feedback can improve force output and time to reach peak force during fast isometric maximal contractions (Jung & Hallbeck, 2004). While real‑time visual feedback is more readily available in laboratory environments, it is the gold‑standard dynamometer used in our study that poses the greater barrier to clinical reproducibility, as this equipment is rarely accessible in routine clinical settings. To acknowledge this limitation, we have added the following sentence to the discussion.

 “Furthermore, it is acknowledged the procedures used in this study rely on equipment and testing conditions that are not routinely available in most clinical settings; therefore, future research should consider not just the reliability of strength testing procedures but also their potential for translation to clinical practice.”

**3.2 RFD is measured in first 200ms. Justification for duration window need to be added. This method has been mentioned as strength of the study.**

 Thank you for this suggestion.

 As already outlined in our Discussion below, we focused on early phase RFD because it reflects initial motor unit recruitment and firing rates—known to be impaired after stroke—and has functional relevance.

 “Our study analysed RFD in the first 200ms, whereas Mentiplay et al. (28) scanned successive 200ms windows to find the peak RFD, a method that excludes movement onset and any associated artefacts or issues with identifying onset. (29) This very early force generation is particularly relevant for people with stroke who have lower motor unit discharge rates (9 , 40) and may be more functionally important than maximal muscle strength or power, especially under circumstances where a rapid response is required (e.g., to prevent falling). (41) Thus, while measuring RFD later in the movement may be more reliable, (28 , 29) this measure may lack ecological validity.”

 To add further clarification, we have added the following justification to the methods section.

 “The 0–200ms window was selected to capture early neural activation and is a commonly used fixed interval for RFD quantification in voluntary contractions (32); furthermore, very short windows (<100 ms) may be less appropriate given the delay in muscle activation post stroke. (8,9)”

**3.3 What is best value for 90% of peak force?, is the time taken in the trial with maximum peak force or trial with minimum time to reach 90% of peak force? In view of looking at ability to produce peak force to tackle real life scenarios requiring quick force production, the best timing must be the shortest time at which 90% force reached.**

 Thank you for this comment which alerted us to the fact that we had incorrectly used the ‘maximum’ value rather than the ‘minimum’ value as the best value for Time to 90% MVC. We have re-analysed this data and revised the results for this metric accordingly. 

 Because the new data for Time to 90% MVC BEST was non-normally distributed, the data for this single outcome was log-transformed. The reliability metrics for log-transformed data are provided in Table 1, but to allow comparability, we have provided the reliability metrics based on raw data at the bottom of the table. The new results for Time to 90% MVC BEST show slightly higher reliability, but as the lower bound of the 95% confidence interval of the ICC is still in the poor range, the revised result does not change the discussion or conclusions of the paper. 

 The manuscript has been updated for this outcome measure.

**References for reviewer responses:**

 Jung, M., & Hallbeck, M. S. (2004). Quantification of the effects of instruction type, verbal encouragement, and visual feedback on static and peak handgrip strength. International Journal of Industrial Ergonomics, 34(5), 367-374. https://doi.org/10.1016/j.ergon.2004.03.008

 McNair, P. J., Depledge, J., Brettkelly, M., & Stanley, S. N. (1996). Verbal encouragement: effects on maximum effort voluntary muscle action. . Br J Sports Med, 30(3), 243-245. https://doi.org/10.1136/bjsm.30.3.243.

 Rendos, N., Harriell, K., Qazi, S., Regis, R., Alipio, T., & Signorile, J. F. (2019). Variations in Verbal Encouragement Modify Isokinetic Performance. . Journal of Strength and Conditioning Research, 33(3), 708-716. https://doi.org/10.1519/JSC.0000000000002998

# Reviewer report: Reviewer response for version 2

**DOI:** 10.5256/f1000research.162800.r286314

**Published:** 2024-06-27

**Authors:** George Koumantakis

**Affiliations:** 1 Physiotherapy, University of West Attica, Athens, Attiki, Greece

I have no further comments to make, as the authors have taken into consideration all my previous suggestions and have meticulously corrected the initial version of the manuscript. All changes and additions are clearly reflected in the current manuscript version. In my opinion, the article can proceed to indexing.

Is the work clearly and accurately presented and does it cite the current literature?

Yes

If applicable, is the statistical analysis and its interpretation appropriate?

Partly

Are all the source data underlying the results available to ensure full reproducibility?

Yes

Is the study design appropriate and is the work technically sound?

Yes

Are the conclusions drawn adequately supported by the results?

Partly

Are sufficient details of methods and analysis provided to allow replication by others?

Yes

Reviewer Expertise:

Reliability, validity, questionnaires, musculoskeletal, physiotherapy, physical therapy, rehabilitation, EMG, functional assessment

I confirm that I have read this submission and believe that I have an appropriate level of expertise to confirm that it is of an acceptable scientific standard.

# Reviewer report: Reviewer response for version 1

**DOI:** 10.5256/f1000research.145331.r202687

**Published:** 2023-09-18

**Authors:** Vassilios Panoutsakopoulos

**Affiliations:** 1 Aristotle University of Thessaloniki, Thessaloniki, Greece

The submitted manuscript comprises a test-retest reliability study. There are some topics that need to be addressed.

**General comments:**

- Address the comments mentioned by Reviewer 1 (George Koumantakis).
- It is recommended to provide, where appropriate, up-to-date references; only a quarter (8/32) of the cited literature was published in the last 5 years.
- State the hypothesis of the study.
- Study Design: it would be of interest to provide results of the unaffected lower limb for comparison.
- Calculation of RFD200ms: what is the rationale to extract this parameter as the force at the time-instant 200 ms after the onset divided by 200? Could the time derivative of the force data provide an alternative insight regarding the RFD?
- Further elaboration of the neuromuscular mechanisms tested in the study could provide additional context in the Discussion.
- Provide the strengths and the limitations of the study.
- The conclusions partly replicate the final part of the Discussion. It is suggested to include recommendations for practical use.

**Specific comments:**

Introduction:

- “the ability to exert force over a short time”: this is the definition of power; it is proposed to delete this phrase.
- “Muscle strength can be measured isokinetically or isometrically”: Clarification is needed.

Materials and Methods:

- Participants: elaborate on the inclusion criteria.
- Measurement procedures: specify the duration of the 4-5 s isometric dorsiflexor MVCs.

Results:

- Table 1: it is suggested to use subscript rather than superscript characters.

Discussion:

- See the respective General Comments.

Is the work clearly and accurately presented and does it cite the current literature?

Partly

If applicable, is the statistical analysis and its interpretation appropriate?

Yes

Are all the source data underlying the results available to ensure full reproducibility?

Yes

Is the study design appropriate and is the work technically sound?

Partly

Are the conclusions drawn adequately supported by the results?

Partly

Are sufficient details of methods and analysis provided to allow replication by others?

Partly

Reviewer Expertise:

sports biomechanics, force measurements, range of motion measurements, aquatic therapy

I confirm that I have read this submission and believe that I have an appropriate level of expertise to confirm that it is of an acceptable scientific standard, however I have significant reservations, as outlined above.

# Response

**Published:** 2024-05-26

**Authors:** Sharon Olsen

**Affiliations:** HRRI, Auckland University of Technology, Auckland, New Zealand

**2.1 Address the comments mentioned by Reviewer 1 (George Koumantakis).**

 Response: We have addressed Reviewer 1’s comments.

**2.2 It is recommended to provide, where appropriate, up-to-date references; only a quarter (8/32) of the cited literature was published in the last 5 years.**

 Response: Thank you for suggesting we review our reference list. We have reviewed the references and amended the manuscript to include some additional references and recent systematic reviews (Azzollini et al., 2021; Chamorro et al., 2017; Dorsch et al., 2021; Kwong et al., 2017; Lomborg et al., 2022; Mentiplay et al., 2015; Mentiplay et al., 2019; Noguchi et al., 2023).

 Whilst many references in the manuscript are older than 5 years, all references are relevant and representative of the small body of literature in this field. Such references are essential to our understanding of tibialis anterior muscle structure and function in healthy adults (Marsh et al., 1981; Ruiz Munoz et al., 2015; Siddiqi et al., 2015) and stroke populations (Fimland et al., 2011; Freire et al., 2015), and provide a review of previous studies investigating the reliability of hemiparetic muscle strength and rapid force production which began in the early 2000s (Eng et al., 2002; Pohl et al., 2000) and has not grown much since (Klarner et al., 2014; Mentiplay et al., 2018).

**2.3 State the hypothesis of the study.**

 Response: We have added the null hypothesis on line 95.

 “The null hypothesis was that the outcome measures are not reliable (intra-class correlation coefficient (ICC) < 0.5).”

**2.4 Study Design: it would be of interest to provide results of the unaffected lower limb for comparison.**

 Response: We agree this would be helpful. However, due to the time already allocation to complete the experimental protocol and the burden it put on people with chronic stroke, it was not deemed feasible to collect data for the unaffected limb in this clinical population.  

**2.5 Calculation of RFD200ms: what is the rationale to extract this parameter as the force at the time-instant 200 ms after the onset divided by 200? Could the time derivative of the force data provide an alternative insight regarding the RFD?**

 Response: In response to the reviewer's query on the rationale for calculating RFD200ms, we chose this method to assess the initial burst of force generation capability, which is crucial for many functional movements in stroke survivors. We also investigated Time to 90% MVC. Both methods offer a straightforward comparison of early force development. Regarding the suggestion to use the time derivative of the force data as an alternative method. Calculating the time derivative of the force/time curve would provide information about the peak RFD in the early part of the contraction. However, peak RFD in small epochs is sensitive to unsystematic variations in the signal and provides peak RFD at an inconsistent time point which is considered a less comprehensive and less standardised approach to measuring RFD (Maffiuletti et al., 2016). We acknowledge that other methods for data processing may offer deeper insights into the rising force/time curve, for example, measuring RFD at multiple time points, or measuring the impulse of the force/time curve (Maffiuletti et al., 2016). However, these methods would require more complex data processing methods, which may be less feasible in clinical contexts. To acknowledge the need to explore alternative methods for data processing, we have made the following amendments to the discussion.

*Line 296*

*“Given our findings, further research is needed to investigate these concerns about the reliability of rapid force production measures in the hemiparetic dorsiflexor muscles. This research should explore alternative methods for data collection and processing (* *Maffiuletti et al., 2016) and seek to identify reliable methods for measuring hemiparetic RFD and muscle power that better account for sources of biological and measurement tool variability.”*

**2.6 Further elaboration of the neuromuscular mechanisms tested in the study could provide additional context in the Discussion.**

 Response: To give greater context, we have added the following information to the introduction.

*Line 9*

*“Rapid force production requires recruitment of a large number of motor units as well as a high motor unit firing frequency, both of which are impaired in the hemiparetic dorsiflexor muscles after stroke.* ^*6*^ *This results from central deficits, which reduce neural input to the motor neuron pool, but is also limited by peripheral changes, such as the reduction in the size of type 2a muscle fibres in the hemiparetic tibialis anterior* *(Noguchi et al., 2023)* *and soft tissue stiffness and spasticity in the antagonist plantarflexor muscles* *(Azzollini et al., 2021)* *.”*

**2.7 Provide the strengths and the limitations of the study.**

**Response: Thank you for this suggestion. We have added a section on strength and limitations to the discussion.**

*Line 302*

*Strengths and limitations*

*“A key strength of this study was the application of an isometric MVC procedure that could be completed by people with more severe stroke. This enabled enrolment of a broad sample with a range of lower limb weakness and functional walking ability, increasing the generalisability of findings to a wider stroke population. Other methodological strengths included positioning of the ankle to optimise dorsiflexion force* *^29^* *and fixation of the toes to reduce measurement variability.* *^23^* *In addition, our approach to measuring RFD enabled evaluation of the very early force production (0-200ms) which has not been evaluated previously in the hemiparetic dorsiflexor muscles. Further research could explore the reliability of other time windows. The key limitation in this study was the sample size, which was below the n=30 recommended for reliability studies ^28^ but comparable with other studies in this field. ^12, 13, 15 ^To address this limitation, we have been cautious with our interpretation of results, and considered both the ICC and its lower bound 95% CI* *^28^* *(Munro, 2005)* *and have provided SEMs to enable comparisons with other literature. However, due to this limitation, it is recommended that the findings generated in this study are confirmed with further research in a larger sample.”*

**2.8 The conclusions partly replicate the final part of the Discussion. It is suggested to include recommendations for practical use.**

 Response: Thank you for this suggestion. Please find below our amended conclusion.

 Line 363

*“This analysis demonstrated excellent between-session reliability for hemiparetic dorsiflexor isometric MVCs. However, other measures of EMG and rapid force production were less reliable, with ICC 95% confidence intervals extending to the poor to moderate range, and SEM percentages between 23-25%. These findings, which utilises gold-standard dynamometry, raise concerns about the reliability of measures of rapid force production in the hemiparetic dorsiflexors muscles. Further research is required to examine reliability in a large sample of people stroke. In the meantime, researchers and clinicians should be cautious when interpreting rapid force production measures of the dorsiflexor muscles when determining the efficacy of stroke rehabilitation interventions. Given the significance of dorsiflexor muscle function to lower limb recovery after stroke, future research should investigate reliable tools for measuring hemiparetic dorsiflexor muscle RFD and muscle power. This will facilitate a greater understanding this aspect of muscle function and enable more targeted rehabilitation."*

**2.9 Introduction: “the ability to exert force over a short time”: this is the definition of power; it is proposed to delete this phrase.**

 Response: It was our intention to define muscle power to ensure this concept was clear at the outset. We have put the definition in brackets to make it clear that this sentence refers to only two concepts.  

 Line 2

*“Ankle dorsiflexor impairments are common after stroke* *^1^* *affecting both muscle strength (the force exerted during a single maximal effort) and muscle power (the ability to exert force over a short time).”*

**2.10 Introduction: “Muscle strength can be measured isokinetically or isometrically”: Clarification is needed.**

 Response: Thank you for this prompt. We have provided clarification in the manuscript as follows.

 Line 65

*“Isokinetic MVCs involve muscle contraction against accommodating resistance through the joints range of movement at a constant velocity, whereas isometric MVCs involve muscle contraction against stationary resistance at a set joint angle* *(Drouin et al., 2004)* *.”*

**2.11 Participants: elaborate on the inclusion criteria.**

 Response: The eligibility criteria have been provided below with some additional clarification.

*“The 15 participants were adults, more than 6 months post stroke, with hemiparesis affecting ankle dorsiflexion movement. The sample size was based on that required for the broader experimental study.* *^9^* *Exclusion criteria were significant cognitive/perceptual/communication deficits, cerebellar stroke, inability to produce ankle dorsiflexor force against the dynamometer, or medical conditions that would impact safety or protocol completion. ^9^”*

 In addition, we have provided further information about the impairment and function of participants in the results section.

*“Participants presented with a range of lower limb weakness, from mild to severe, and used a variety of outdoor mobility aids (unaided n = 4, quad or walking stick n = 5, walking frame n = 2, wheelchair n = 2) suggesting a range of walking abilities.”*

**2.12 Measurement procedures: specify the duration of the 4-5 s isometric dorsiflexor MVCs.**

 Response: The duration was 4 to 5 seconds. We have written this more clearly in the manuscript.

*Line 117*

*“Following two submaximal practices, participants performed three isometric dorsiflexor MVCs; each lasted a duration of 4 to 5 seconds and a 2-minute rest was given between each MVC.”*

**2.13 Results Table 1: it is suggested to use subscript rather than superscript characters.**

 Response: We have changed these outcomes to subscript as suggested.

# Reviewer report: Reviewer response for version 1

**DOI:** 10.5256/f1000research.145331.r191140

**Published:** 2023-08-11

**Authors:** George Koumantakis

**Affiliations:** 1 Physiotherapy, University of West Attica, Athens, Attiki, Greece

This is a well-conducted test-retest reliability study. The authors know the correct statistical methods to report such a study. Minor issues are present, and these are highlighted to improve this work.

 The authors should mention in their Abstract the respective SEM values as well.

 The authors correctly make adequate links to the clinical utility of this study, as strength and rate of force development in the ankle dorsiflexors form part of participants’ gait improvement post-stroke.

 Under ‘Participants’, the sample size (n=15), which eventually was n=13, is not adequately explained. Koo & Li (2016), pg. 158, suggest a sample of n=30 as adequate for a reliability study. The best you can do is add this to the limitations of your study.

 It is unclear how many raters were in this study; please clarify.

 Correct the characterization of ICCs, based not only on the lower limit of the ICC 95% CI but on the whole range of the CI, as suggested by Koo and Li (2016), pg. 161. Please correct throughout.

 The EMG acquisition and analysis are correctly reported.

Is the work clearly and accurately presented and does it cite the current literature?

Yes

If applicable, is the statistical analysis and its interpretation appropriate?

Partly

Are all the source data underlying the results available to ensure full reproducibility?

Yes

Is the study design appropriate and is the work technically sound?

Yes

Are the conclusions drawn adequately supported by the results?

Partly

Are sufficient details of methods and analysis provided to allow replication by others?

Yes

Reviewer Expertise:

Reliability, validity, questionnaires, musculoskeletal, physiotherapy, physical therapy, rehabilitation, EMG, functional assessment

I confirm that I have read this submission and believe that I have an appropriate level of expertise to confirm that it is of an acceptable scientific standard, however I have significant reservations, as outlined above.

# Response

**Published:** 2024-05-26

**Authors:** Sharon Olsen

**Affiliations:** HRRI, Auckland University of Technology, Auckland, New Zealand

**1.1 This is a well-conducted test-retest reliability study. The authors know the correct statistical methods to report such a study. Minor issues are present, and these are highlighted to improve this work**

 Response: Thank you for your encouraging feedback.

**1.2 The authors should mention in their Abstract the respective SEM values as well.**

 Response: Thank you for this suggestion. We have added the SEM% values to the abstract results below.

*Abstract results: “Reliability was higher when analysing the mean of three trials rather than the best of three trials. There was excellent reliability for isometric dorsiflexor MVC (ICC 0.97 [95% CI 0.92, 0.99], SEM% 7%). However, for other outcomes, while the ICC indicated good reliability, the lower bound of the 95% confidence interval of the ICC fell in the moderate range for TA EMG (ICC 0.86 [95% CI 0.60, 0.96], SEM% 25%) and time to reach 90% MVC (ICC 0.8 [95% CI 0.53, 0.93], SEM% 23%) and in the poor range for dorsiflexor RFD200ms (ICC 0.79 [95% CI 0.48, 0.92], SEM% 24%).”*

**1.3 The authors correctly make adequate links to the clinical utility of this study, as strength and rate of force development in the ankle dorsiflexors form part of participants’ gait improvement post-stroke.**

 Response: Thank you for this encouraging feedback.

**1.4 Under ‘Participants’, the sample size (n=15), which eventually was n=13, is not adequately explained. Koo & Li (2016), pg. 158, suggest a sample of n=30 as adequate for a reliability study. The best you can do is add this to the limitations of your study.**

 Response:  Thank you for raising this concern. While other reliability studies in this field of stroke research have been conducted with similarly small samples (Eng et al., 2002; Klarner et al., 2014; Pohl et al., 2000), we agree that there is a risk of being underpowered. Therefore, we have amended the manuscript and discussed the sample size in the new limitations section copied below and made recommendations for further research in a larger sample.

*Strengths and limitations: Line 302*

*…"The key limitation in this study was the sample size, which was below the n=30 recommended for reliability studies ^28^ but comparable with other studies in this field. ^12, 13, 15^ To address this limitation, we have been cautious with our interpretation of results, and considered both the ICC and its lower bound 95% CI* *(Koo & Li, 2016; Munro, 2005)* *and have provided SEMs to enable comparisons with other literature. However, due to this limitation, it is recommended that the findings generated in this study are confirmed with further research in a larger sample.”*

 In addition, we have also provided sample sizes of other research in the discussion to aid in the interpretation.

*Line 262  "... Our MVC reliability results were superior to the isometric MVC results of Klarner and colleagues who found moderate between-session reliability (ICC 0.71) for hemiparetic dorsiflexor MVCs over *three sessions, with a similar sample size (n=12). ^15 "^

*Line 267 "...a* *s with the MVC data, our TA EMG data appeared more reliable than that previously reported (ICC 0.67) in sample of 12 people with chronic stroke.* ^15 ^”

 Furthermore, we have provided further explanation about the 2 dropouts in the results section.

*Line 156 "Data for two participants were excluded due to failure to correctly complete the protocol; this was because one participant was not able to consistently follow the task instructions and another participant was observed falling asleep during the protocol.”*

 In addition to these amendments related to the sample size and study power, we have revised our approach to the interpretation of the ICCs to take a more conservative approach – these amendments are detailed in question 1.6 which raised the issue of ICC interpretation. Please see 1.6.

**1.5 It is unclear how many raters were in this study; please clarify.**

 Response: Thank you for raising this question. This paper investigates between-session reliability, rather than inter-rater reliability. The aim of the paper is stated at the end of the introduction. “This analysis aimed to determine the between-session reliability of isometric ankle dorsiflexor MVC, ankle dorsiflexor muscle RFD in the first 200ms (RFD200ms), time to reach 90% peak force, and TA EMG, in people with chronic stroke.”

 In response to your question, we have clarified in the manuscript that the same researcher provided verbal instructions at the two measurement sessions, and that the visual inspection of data was completed by a single researcher.

 Line 119

*“Participants were instructed to “pull as fast and hard as possible” and received loud verbal encouragement and real-time visual feedback. Instructions were provided by the same researcher at both sessions.”*

 Line 131

*“Movement onset was automatically identified where the signal exceeded the mean baseline signal by 3 SDs, and then confirmed visually by a single researcher. The baseline window and the onset threshold could be individualised by the researcher to ensure the onset was identified correctly for each contraction.”*

**1.6 Correct the characterization of ICCs, based not only on the lower limit of the ICC 95% CI but on the whole range of the CI, as suggested by Koo and Li (2016), pg. 161. Please correct throughout.**

 Response: Thank you for raising this. We had chosen to interpret ICCs based on the lower bound of the 95% CI as a conservative approach which considers the sample is not a random sample of the population (Munro, 2005). However, given our sample was small (which we have discussed in 1.4 above), we agree that the full range should be considered and have amended the manuscript to take a more conservative approach to interpretation of the findings and clarified when we are interpreting the ICC or its lower bound 95% confidence interval. Relevant amendments to the abstract, discussion and conclusion are copied below.

*Abstract*

*“Results: …There was excellent reliability for isometric dorsiflexor MVC (ICC 0.97 [95% CI 0.92-0.99], SEM% 7%). However, for other outcomes, while the ICC indicated good reliability, the lower bound of the 95% confidence interval of the ICC fell in the moderate range for TA EMG (ICC 0.86 [95% CI 0.60-0.96], SEM% 25%) and time to reach 90% MVC (ICC 0.8 [95% CI 0.53-0.93], SEM% 23%) and in the poor range for dorsiflexor RFD200ms (ICC 0.79 [95% CI 0.48-0.92], SEM% 24%).*

*Conclusion: The findings raise concerns about the reliability of measures of rapid force production in the dorsiflexor muscles after stroke. Given the functional significance of the ankle dorsiflexors, larger studies should be conducted to further investigate these concerns and explore reliable methods for measuring rapid force production in the hemiparetic dorsiflexor muscles.”*

*Results - Reliability analysis*

*The reliability analysis is reported in Table 1. MVC measures demonstrated excellent reliability, with the mean of three trials displaying slightly higher reliability (ICC 0.97 [95% CI 0.92, 0.99]) than the best of three trials (ICC 0.97 [95% CI 0.90, 0.99]). For TA EMG data, the ICCs were in the good range but the lower bound 95% CIs were in the moderate range (ICC 0.86 [95% CI 0.60, 0.06]). For measures of rapid force production, when using the mean of three trials, the Time to 90% MVC and RFD200ms had ICCs in the good range, but the lower bound 95% CIs were in the moderate range for Time to 90% MVC (ICC 0.80 [95% CI 0.53, 0.93]) and in the poor range for RFD200ms (ICC 0.0.79 [95% CI 0.48, 0.92]). Both measures of rapid force production demonstrated very low lower-bound CIs when only the best trial was analysed (Table 1).*

 Please note we have removed the interpretation column in Table 1, and instead provided this in the text to enable interpretation of both the ICC and its confidence interval.

*Discussion*

*Line 267*

*“…Our reliability findings for TA EMG, which represent motor unit recruitment at the peak of the MVC, demonstrated lower reliability, with a good ICC and the lower bound 95% CI in the moderate range (TA EMG _MEAN_ ICC 0.86 [95% CI 0.60, 0.96).* *Alongside an SEM% of 23-25%, this suggests that TA EMG is prone to greater biological and/or measurement variability than peak force measures.”*

*Line 274*

*“…This study is also the first to report on the reliability of RFD or rapid force production of the hemiparetic dorsiflexor muscles using a rigid dynamometer. While the ICCs were in the good range when three trials were analysed, the lower bound of the 95% CI of the ICCs indicated reliability could be only moderate for Time to 90% MVC _MEAN_ (ICC 0.80 [95% CI 0.53, 0.93]) and poor for RFD200ms _MEAN_ (ICC 0.79 [95% CI 0.48, 0.92]).”*

*Line 296*

*“Given our findings, further research is needed to investigate these concerns about the reliability of rapid force production measures in the hemiparetic dorsiflexor muscles. This research should explore alternative methods for data collection and processing (* *Maffiuletti et al., 2016) and seek to identify reliable methods for measuring hemiparetic RFD and muscle power that better account for sources of biological and measurement tool variability.”*

*Conclusions*

*“This analysis demonstrated excellent between-session reliability for hemiparetic dorsiflexor isometric MVCs. However, other measures of EMG and rapid force production were less reliable, with ICC 95% confidence intervals extending to the poor to moderate range, and SEM percentages between 23-25%. These findings, which utilise gold-standard dynamometry, raise concerns about the reliability of measures of rapid force production in the hemiparetic dorsiflexors muscles. Further research is required to examine reliability in a large sample of people stroke. In the meantime, researchers and clinicians should be cautious when interpreting rapid force production measures of the dorsiflexor muscles when determining the efficacy of stroke rehabilitation interventions. Given the significance of dorsiflexor muscle function to lower limb recovery after stroke, future research should investigate reliable tools for measuring hemiparetic dorsiflexor muscle RFD and muscle power. This will facilitate a greater understanding this aspect of muscle function and enable more targeted rehabilitation.”*

**1.7 The EMG acquisition and analysis are correctly reported.**

 Response: Thank you.

**1.8 Are the conclusions drawn adequately supported by the results? Partly**

 Response: Thank you for indicating this. We have amended our conclusion with a more cautious approach to interpretation and based on Reviewer 2’s feedback, we have added implications for researchers and clinicians. The conclusions have been copied above in 1.6.
